# Nudibranch predation boosts sponge silicon cycling

**DOI:** 10.1038/s41598-023-27411-y

**Published:** 2023-01-20

**Authors:** María López-Acosta, Clémence Potel, Morgane Gallinari, Fiz F. Pérez, Aude Leynaert

**Affiliations:** 1 Instituto de Investigaciones Marinas (IIM), CSIC, C/Eduardo Cabello 6, 36208 Vigo, Spain; 2 Laboratoire des Sciences de l’Environnement Marin, UMR 6539, Institut Universitaire Européen de la Mer, Technopôle Brest-Iroise, 29280 Plouzané, France; 3 Learning Planet Institute, Université de Paris, 8 bis rue Charles V, 75004 Paris, France

**Keywords:** Biogeochemistry, Ecology

## Abstract

Diatoms play a key role in the marine silica cycle, but recent studies have shown that sponges can also have an important effect on this dynamic. They accumulate large stocks of biogenic silica within their bodies over long periods, which are thought to vary little on an intra-annual scale. The observation of an abrupt decline in sponge biomass in parallel with large increases in abundance of a spongivorous nudibranch (*Doris verrucosa*) led us to conduct a year-long study on the effect of nudibranch predation on the silicon budget of a sponge (*Hymeniacidon perlevis*) population. After 5 months of predation, the abundance of sponge individuals did not change but their biomass decreased by 95%, of which 48% was explained by nudibranch predation. About 97% of sponge spicules ingested by nudibranchs while feeding was excreted, most of them unbroken, implying a high rate of sponge silica deposition in the surrounding sediments. After predation, sponges partially recovered their biomass stocks within 7 months. This involved a rapid growth rate and large assimilation of dissolved silicon. Surprisingly, the highest rates of silicon absorption occurred when dissolved silicon concentration in seawater was minimal (< 1.5 µM). These findings suggest that the annual sponge predation-recovery cycle triggers unprecedented intra-annual changes in sponge silicon stocks and boosts the cycling of this nutrient. They also highlight the need for intra-annual data collection to understand the dynamics and resilience of sponge ecosystem functioning.

## Introduction

Silicon is a key nutrient in the ocean necessary for a variety of marine organisms to build their siliceous skeletons and grow. These organisms, known as silicifiers, include organisms of different trophic levels and ecological relevance, such as diatoms, silicoflagellates, most species of rhizarians and sponges, and some species of choanoflagellates^1^. Among them, diatoms and sponges are the most abundant pelagic and benthic silicifiers, respectively. These organisms play important ecological roles in marine ecosystems. Diatoms account for about 40% of ocean primary productivity and export of particulate carbon from the surface to the deep ocean^2,3^. Sponges contribute to the recycling of nutrients to higher trophic levels through benthic-pelagic coupling and to increasing marine biodiversity of micro- and macro-organisms^4,5^. Thus, silicon is a critical resource for silicifiers, which can be important in sustaining marine biodiversity and food webs with ultimate influences on human populations^6,7^.

Among silicifiers, sponges are the only group of metazoans (i.e., animals). They are multicellular, heterotrophic organisms with long lifespans, which can last from years to, perhaps, millennia^8,9^. Sponges are common components of marine benthic fauna across global oceans, being numerically abundant and biomass dominant in many marine ecosystems from polar to equatorial latitudes^10^. As in other silicifiers, sponges consume dissolved silicon from seawater to build their skeletons made of biogenic silica (i.e., opal). These skeletons are more resistant to dissolution than those of other silicifiers, for reasons yet to be determined^11^. Their biological and skeletal characteristics make siliceous sponges major silicon sinks in marine ecosystems, accumulating stocks of biogenic silica within their tissues and in the sediments beneath them^11,12^, and recycling their silicon at significantly slower rates (200–1000 times slower) than that of other short-lived, unicellular silicifiers such as diatoms^13^. Still, in areas where they form large aggregations (e.g., sponge grounds), sponge silicon cycling can enrich dissolved silicon demersal layers on an oceanographic scale^14^.

The dynamics of sponge populations depend on recruitment, longevity, space competition, and predation^15^. In particular, predation can affect sponge community structure and ecosystem processes^16,17^. Sponge predation has been reported on tropical and subtropical sponge coral reefs^18–20^, mesophotic sponge reefs^21,22^, and at polar latitudes from intertidal to deep-sea environments^17,23^. Spongivory, i.e., feeding on sponges, has been described in some species of mollusks, echinoderms, fishes, and turtles^18–21^. Among mollusks, dorid nudibranchs (Order Nudibranchia, Family Dorididae) are one of the most diverse groups of sponge predators. Most of them are strict spongivores with radulae adapted to the specific sponge skeletal organization on which they feed^24^. Many also have a cryptic coloration with their prey^25^. This high predator–prey specificity implies that most species of dorid nudibranchs feed on one or few sponge species, which they sometimes overgraze^23,26,27^.

Sponges possess a remarkable capacity of regeneration, regardless of their phylogeny or species traits^28^. When they are injured or lose a part of their body, a battery of molecular and cellular processes is activated to repair the wound^29^, with regeneration rates much higher than the normal growth rate^28^. Here we hypothesize that sponge predation and subsequent regeneration processes affect sponge silicon cycling. To test this hypothesis, we surveyed for one year the population of the sponge species *Hymeniacidon perlevis*, a globally-distributed species^30^ that dominates the sponge community of the Bay of Brest (France). In this shallow-water ecosystem, field observations indicate that the sponge *H. perlevis* is subjected annually to predation by the nudibranch *Doris verrucosa*. To test the effects of predation on the sponge, we quantified the rate of predation by *D. verrucosa*, the rate of sponge silicon export during predation, and the rate of silicon production by *H. perlevis* during recovery from predation.

## Materials and methods

### Study species

**Figure 1:**
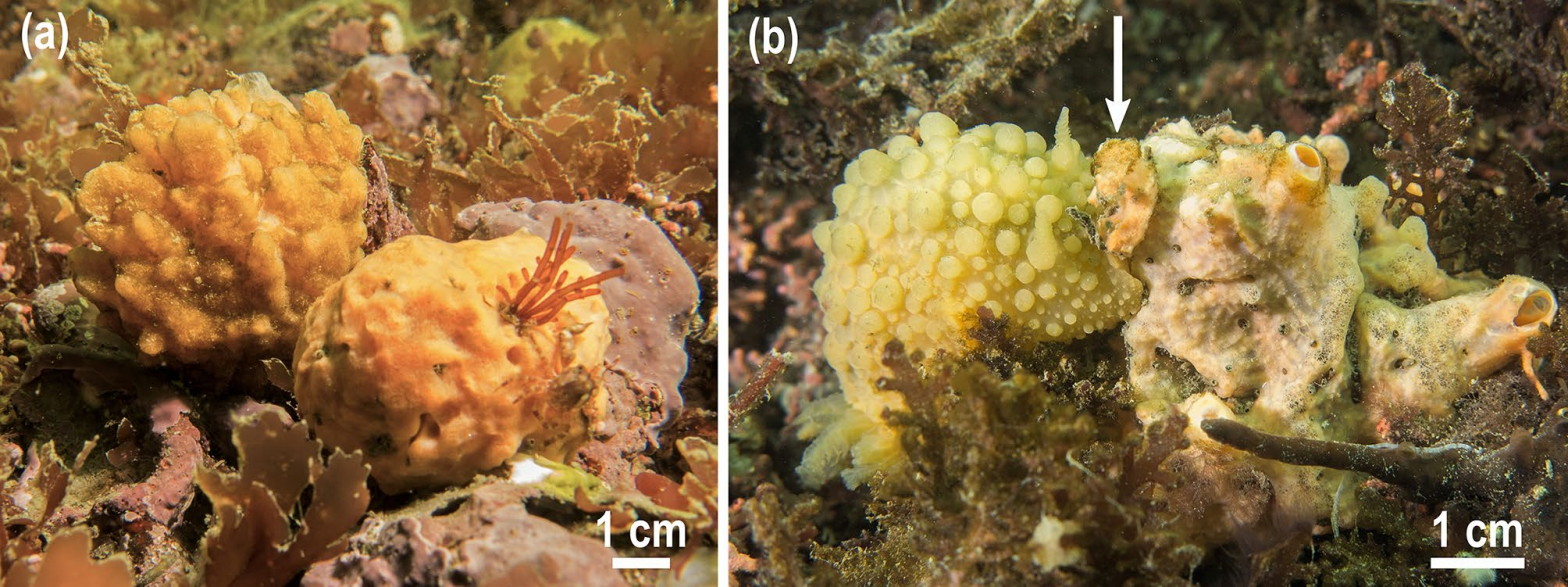
(**a**) View of two individuals of *Hymeniacidon perlevis* growing on the maerl beds of the Bay of Brest (France). (**b**) An individual of *Doris verrucosa* (left) feeds on the sponge *H. perlevis* (right). The arrow indicates the wound made by the nudibranch when feeding on the sponge, showing the inner tissues of the sponge of a darker orange color than the epidermis.

The sponge *Hymeniacidon perlevis* and the nudibranch *Doris verrucosa* coexist in the maerl bed of Lomergat (48° 17.197′ N; 4° 21.279′ W), a shallow-water ecosystem (depth = 3–11 m) located in the Bay of Brest (France). This habitat occupies a small area of the Bay (1.44 km^2^ of the total 133.13 km^2^, i.e., 1.1%). In this ecosystem, assemblages of maerl (coralline red algae) serve as substrate for a highly diverse benthic community, including more than 20 species of sponges^13^. Among them, *H. perlevis* (Fig. 1a) is the dominant species in terms of both abundance (53%) and biomass (64%)^13^. It has a moderate content of siliceous skeleton derived from the production of a single, mid-size (175–475 µm long) type of needle-like silica spicule, which is homogenously distributed throughout the sponge body^31^.

The study area is also habitat for the dorid nudibranch *D. verrucosa*. This spongivorous nudibranch lives from the intertidal zone to 15 m depth along the Atlantic coast of Europe and the Mediterranean Sea, and is a strict spongivore^32^. This species feeds mainly on the sponge *H. perlevis*, although it has occasionally been reported feeding on the sponges *Halichondria panicea* and *Suberites carnosus* in the Spanish Atlantic coast and the Mediterranean Sea^33,34^. These three sponge species occur in the Bay of Brest, but we have only found *D. verrucosa* feeding on *H. perlevis* (Fig. 1b), and confirmed this observation by analysis of its fecal pellets.

### Prey abundance

The abundance and biomass of *H. perlevis* were surveyed during scuba diving for one year (June 2020–June 2021) using randomly positioned 1 × 1 m quadrats (n = 71). Four surveys were conducted: June 2020, to assess the sponge population before predation; November 2020, to evaluate the sponge population after predation; and April and June 2021, to assess the sponge population recovery. During field surveys, each individual of *H. perlevis* found within the quadrats was counted and its volume was measured. To determine the sponge volume, the body shape of each individual was approximated to one or a sum of several geometric figures (e.g., sphere, cylinder, cube) and the linear parameters needed to calculate the volume (length, width, height, diameter) were measured in situ with a ruler. Differences in sponge abundance and biomass between sampling times (i.e., June 2020, November 2020, April 2021 and June 2021) were examined using one-way Kruskal–Wallis analysis after the normality test (Shapiro–Wilk) failed. When significant differences were reported, post-hoc pairwise multiple comparisons between groups were conducted using the non-parametric Dunn’s test. Statistical analyses—with a significance level of 0.05—and associated data visualization with box plots were performed using the statistical software Sigmaplot 14.5 (Systat Software Inc.).

At each sampling time, 5 individuals of *H. perlevis* were sampled to determine the specific silicon content. Sponge tissue from each individual was sampled (~ 1–5 mL) and then dried at 60ºC to constant dry weight (g). Each dried sample was digested for 5 h in 20 mL of hydrofluoric acid at 10% (2.9 N). No interference from lithogenic silicon was expected because the samples only contained sponge tissue. After complete digestion, samples in hydrofluoric acid were neutralized with a saturated solution of boric acid (H_3_BO_3_, 60 g L^−1^) and dissolved silicon was measured according to the molybdate-blue method by colorimetry on a SEAL Analytical AA3 HR auto-analyzer^35^. Differences in sponge silicon content between sampling times were examined using one-way ANOVA. Data on the specific silicon content and sponge biomass measured in situ after predation (i.e., November 2020, April and June 2021) were used to estimate the rate of sponge silicon production by *H. perlevis* during population recovery when predators were absent.

### Predator abundance

The abundance and biomass of *D. verrucosa* was quantified using 8 transects of 25 m × 2 m during scuba diving in late June and early July 2020 (total area = 400 m^2^) during estimated peak abundance. *Doris verrucosa* were counted and measured (length in mm), and feeding activity was recorded. We inferred that nudibranchs were feeding when they were immobile on a sponge.

Nudibranch shape was approximated to that of an ellipsoid (Eq. 1):

1 $$Nudibranch\,\, volume \,\,\left(mL\right)=\frac{4}{3}\times \pi \times a\times b\times c$$

where ‘*a*’, ‘*b*’ and ‘*c*’ are the radii (in cm) of each ellipsoid axes, corresponding to half of the length, width and height of the animal, respectively. These parameters plus wet weight (g, measured after blotting with paper towels) were measured in 36 nudibranchs in the laboratory. These measurements were used to generate regressions that were used to estimate nudibranch biomass and volume from the measurements taken in the field.

### In situ nudibranch feeding experiments

**Figure 2:**
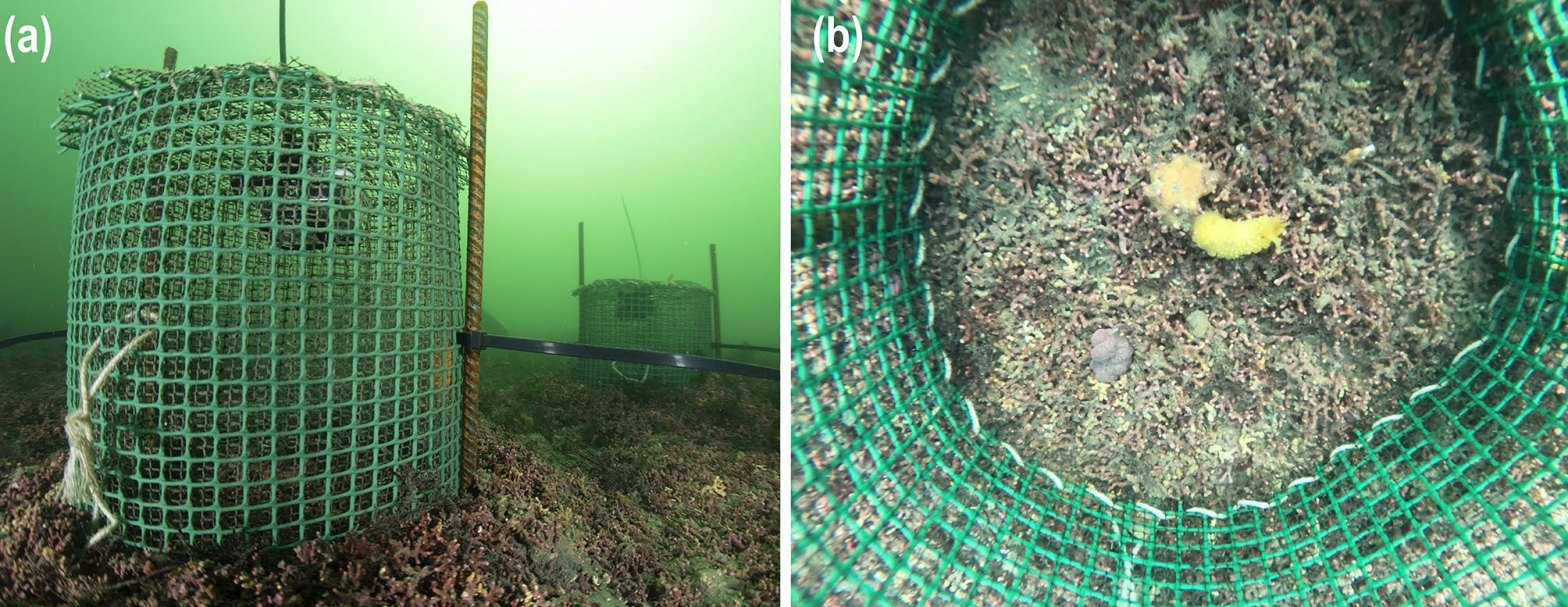
(**a**) In situ experimental setup to determine nudibranch feeding rates on the sponge *Hymeniacidon perlevis* in the maerl bed of Lomergat (Bay of Brest, France). (**b**) Top-down view of the inside of an incubation cage. The nudibranch *Doris verrucosa* approaches to eat the sponge *H. perlevis* after 30 min of incubation. Time lapse of the in situ incubation is available as Video S1.

Individual predation rates of *D. verrucosa* on *H. perlevis* were determined in situ. We constructed cylindrical cages 20-cm high by 25-cm in diameter using two nets of different mesh size that were stitched together with a nylon line (Fig. 2). The smaller net had a 2 × 2 mm mesh to prevent nudibranchs from escaping. The cages were open on the bottom and were fixed to the seafloor with steel rebar (Fig. 2a). Twelve trials were performed in October 2020. In nine cages, one individual of *H. perlevis* and one *D. verrucosa* were held together for 24 h (Fig. 2b). Three control cages were predator-free. Sponge and nudibranch volumes were measured before and after each trial as explained above for field surveys. Individual predation rates of *D. verrucosa* on *H. perlevis* were calculated as the difference between sponge volume at the beginning and the end of each experiment normalized by nudibranch biomass and trial length using Eq. (2).

2 $$Predation\,\, rate=\frac{({Sponge\,\, volume}_{{t}_{0}}-{ Sponge\,\, volume}_{{t}_{f}})}{Nudibranch \,\,volume \times time \,\,of \,\,incubation}$$

### Sponge silicon in nudibranch feces

Individual nudibranch fecal output was determined in the laboratory using a different set of nudibranchs and sponges. Sponges and nudibranchs were acclimated separately for 5 days before the experiment, a period sufficient to allow the nudibranchs to clear their digestive tract from previous feeding^26^. Eight trials were performed, each including a different nudibranch with a sponge for 72 h in a 50 L aquarium filled with running seawater from the Bay of Brest. To exclude particles from the inflowing water that could settle to the bottom and be erroneously included as nudibranch feces, the inflow was filtered with a 1 µm pore size filter. The constant inflow ensured the renewal of the water inside each aquarium several times a day.

Feces were sampled with a pipette once a day during each trial and for 72 h after the trial, when fecal production ended. Before further treatment, feces were examined under an optical microscopy with a digital camera (Leica DM2500) to determine their general appearance and the presence of entire or fragmented sponge skeletal pieces (spicules). Samples were then dried at 60 °C to constant dry weight (g). To determine the sponge silicon content in nudibranch feces, three subsamples (~ 8 mg) from each aquarium were digested for 24 h in 40 mL of NaOH 0.2 M to ensure total digestion of sponge silicon^11^. Dissolved silicon was measured using the molybdate-blue method by colorimetry on a SEAL Analytical AA3 HR auto-analyzer^35^. To correct the proportion of lithogenic silica that dissolved at the same time as sponge silicon (i.e., biogenic silica), we quantified the aluminum content. Dissolved aluminum was determined according to the fluorimetric method using lumogallion^36^. Chemical analysis of aluminum and silicon in the fecal pellets of nudibranchs revealed that the aluminum:silicon ratio was < 1.5%, indicating that lithogenic silica in nudibranch feces was negligible. We therefore assumed that the silicon measured in the feces was entirely sponge silicon.

To determine the percentage of sponge skeleton ingested that was excreted in nudibranch feces, the amount of sponge eaten by each nudibranch in the aquaria was measured using Eq. (2), as explained above. Note that this ingestion rate cannot be compared to that measured in situ as assayed nudibranchs in the laboratory were starved for 5 days before incubation. Also, three samples of sponge tissue (~ 1 mL) of each incubated sponge were sampled to determine the silicon content, which were dried at 60 °C to constant dry weight (g) before silicon determination. Silicon content was determined using hydrofluoric acid digestion as for the samples from the sponge field survey (see above). The percentage of ingested sponge skeleton that was excreted in nudibranch feces was determined from the amount of silicon that each nudibranch ingested when feeding and compared with that found in feces.

Individual rates of sponge feeding and fecal production of *D. verrucosa* and the abundance of nudibranchs and sponges in the natural habitat were used to estimate the amount of sponge silicon deposited on the habitat sediments through nudibranch feces during the period of the year the nudibranchs were present (i.e., mid-June to mid-November).

### Sponge silicon in non-predated sponges

Silicon fluxes of non-predated sponges were estimated from data in the literature to compare with those measured in this study. Silicon production in non-predated sponges was estimated from the species silicon consumption kinetics^37^ at the concentration of dissolved silicon in the bottom waters of the Bay of Brest, which is measured weekly by the SOMLIT-Lanvéoc Observation Service. We scaled this measure to the area of study using the sponge biomass (mL m^−2^) measured in situ during this study. Silicon deposition in non-predated sponges was estimated from the average sponge silicon deposition measured in field (0.9 ± 0.2 g Si m^−2^ y^−1^)^13^ and by assuming that the contribution of sponge silicon by *H. perlevis* should be proportional to its standing stock in the sponge community per m^2^, which is 65.5 ± 21.0%. Silicon standing stock of non-predated sponges during the year was estimated by adding silicon production to the silicon standing stock measured in situ in mid-June 2020 and subtracting the silicon deposition. To compare silicon fluxes of predated and non-predated sponges, the stock-specific flux rates were calculated by normalizing the flux rate (either for production or deposition) by the sponge silicon stock. See Supplementary Information 1 for metadata and detailed calculations.

## Results

### Prey and predator abundance

**Figure 3:**
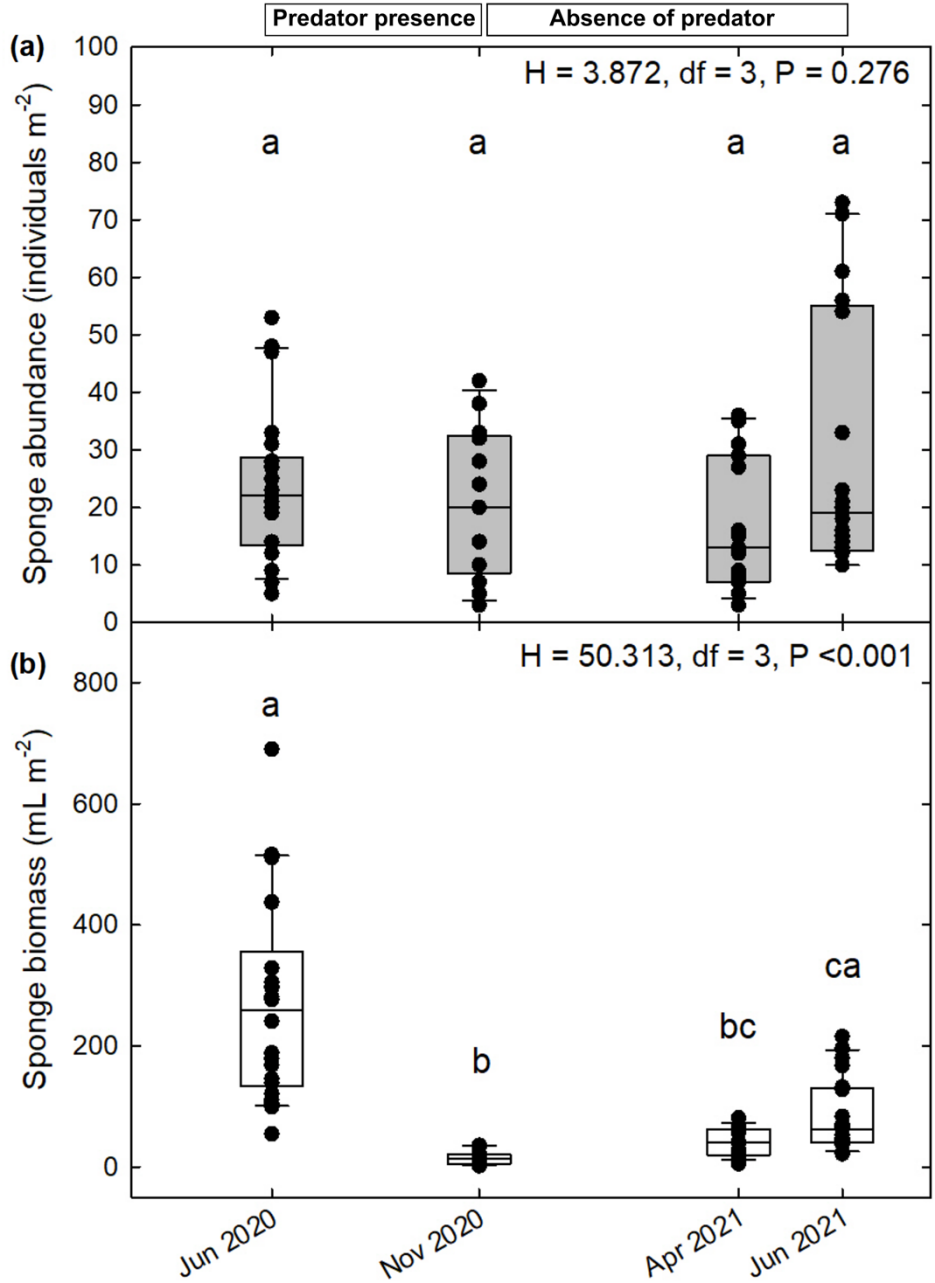
Changes in (**a**) abundance and (**b**) biomass of the sponge *Hymeniacidon perlevis* during an annual cycle. Boxplots and individual data points are indicated for each sampling date. The number of 1 × 1 quadrats analyzed on each sampling date is n_Jun 2020_ = 22, n_Nov 2020_ = 13, n_Apr 2021_ = 15 and n_Jun 2021_ = 21. Significant between-season differences of (**a**) abundance (individuals m^−2^) and (**b**) biomass (mL m^−2^) of the sponge population of *H. perlevis* are indicated with different letters according to the results of Kruskal–Wallis analysis and the post-hoc pairwise Dunn’s tests. The spongivorous nudibranch *Doris verrucosa* was present from mid-June to mid-November in the area of study. Mean (± SD) values for sponge abundance and biomass, as well as metadata, are available in Supplementary Information 1.

The field survey revealed that the abundance of *Hymeniacidon perlevis* remained stable throughout the annual cycle, with no differences between sampling times (H = 3.872, df = 3, p = 0.276; Fig. 3a). Mean abundance (± SD) of *H. perlevis* at the study area was 24 ± 17 individuals m^−2^ (n = 71). In contrast, sponge biomass changed throughout the annual cycle (H = 50.313, df = 3, p < 0.001; Fig. 3b). The highest sponge biomass was recorded in mid-June 2020 (270 ± 164 mL of sponge m^−2^) before predation started and the lowest sponge biomass record was in mid-November 2020 (15 ± 11 mL of sponge m^−2^) after the predation period. This implied a loss of sponge biomass from mid-June to mid-November of 95 ± 1%. Five months after the end of the predation season (mid-April 2021), the sponge biomass was 42 ± 23 mL m^−2^, and 2 months later (mid-June 2021), it was 84 ± 60 mL m^−2^. This suggests that before predation started the following June, 31 ± 5% of the initial biomass of *H. perlevis* was recovered (Fig. 3b). The variation associated (i.e., SD) with the sponge abundance and biomass normalized per m^2^ derived mainly from their patchy spatial distribution and inter-individual size variability (Fig. 3).

Adults of *D. verrucosa* were only present in the study area from mid-June to mid-November. Nudibranch abundance and size (length) were measured during the estimated population peak. A total of 271 individuals of *Doris verrucosa* were recorded in the 400 m^2^ studied, which were frequently found eating in groups on a single sponge (up to 8 nudibranchs on the same sponge). Abundance of *D. verrucosa* averaged 0.7 ± 0.4 individuals m^−2^, with a maximum of 9 nudibranchs m^-2^. Nudibranch length ranged from 2 to 8 cm, with a mean length of 4.4 ± 1.3 cm. Individual length values were used to estimate nudibranch biomass using regressions between length, volume and wet weight. The linear regression between nudibranch length and volume was: Volume (mL) = − 3.6337 + (2.0761 × length (cm)) (n = 36, R^2^ = 0.64, p < 0.001), and the regression between wet weight and volume was: Wet weight (g) = 0.9646 + (1.0360 × Volume (mL)) (n = 36, R^2^ = 0.82, p < 0.001). Average nudibranch volume was 3.7 ± 2.5 mL m^−2^ and wet-weight was 4.5 ± 2.9 g m^−2^. Nudibranch volume was used for rate normalization because it facilitated comparison with field measurements. Data on nudibranch wet weights are available in Supplementary Information 1.

Silicon content of *H. perlevis* per unit of biomass was determined throughout the year and no differences between seasons were found (F = 0.209, df = 3, p = 0.888). This indicates that the skeleton content does not vary whether the sponge has been predated or is regenerating its biomass after predation. Mean skeleton content (i.e., biogenic silica) per dry weight of *H. perlevis* was 20.9 ± 5.6%.

Silicon production of *H. perlevis* during post-predation regeneration (i.e., mid-November to mid-June) was estimated by combining the species skeletal content and sponge biomass records measured after predation (Table 1). The biomass of *H. perlevis* increased by 27.7 ± 11.8 mL m^−2^ from mid-November to mid-April, and by 42.0 ± 37.2 mL m^−2^ from mid-April to mid-June, which in terms of silicon is 0.3 ± 0.2 and 0.4 ± 0.5 g Si m^−2^, respectively. All together, silicon production of the sponge *H. perlevis* after predation (i.e., mid-November to mid-June) was 0.7 ± 0.7 g Si m^−2^.

**Table 1:** Summary of average (± SD) growth and silicon production rates of *Hymeniacidon perlevis* during regeneration.

Period of time	N days	Biomass increase (mL m^−2^)	Si content (g Si mL^−1^)	Sponge Si production (g Si m^−2^)
Nov–April	151	27.7 (± 11.8)	9.8 (± 2.6)	0.3 (± 0.2)
April–June	61	42.0 (± 37.2)	9.8 (± 2.6)	0.4 (± 0.5)
Nov–June	212	69.7 (± 49.0)	9.8 (± 2.6)	0.7 (± 0.7)

The total regeneration period (mid-November to mid-June, 212 days) was explored in two intervals: (1) from mid-November to mid-April, and (2) from mid-April to mid-June. Metadata and tracked calculations are available in Supplementary Information 1.

### Nudibranch predation rates

In the field, individuals of *D. verrucosa* consumed from 0.24 to 1.60 mL of *H. perlevis* per day. Such consumption resulted in a mean normalized predation rate of 0.23 ± 0.11 mL sponge d^−1^ mL^−1^ nudibranch. Sponges incubated without predators (controls; n = 3) showed no change in volume.

Individual predation rates measured in situ were extrapolated to the natural habitat using field estimates of *D. verrucosa* abundance (see above). This resulted in a mean total predation rate of 0.84 ± 0.97 mL sponge d^−1^ m^−2^. The large variation associated (i.e., standard deviation) with the predation rate normalized per m^2^ mainly derived from the patchy spatial distribution of nudibranchs. By extrapolating this predation rate to the period *D. verrucosa* was actively feeding (153 days, mid-June to mid-November), the predation rate on *H. perlevis* by *D. verrucosa* was estimated to be 128.0 ± 148.7 mL sponge m^−2^. At this rate, the predation activity of *D. verrucosa* would be responsible for 48 ± 33% of the sponge population decline measured over the predation period.

### Sponge silicon in nudibranch feces

**Figure 4:**
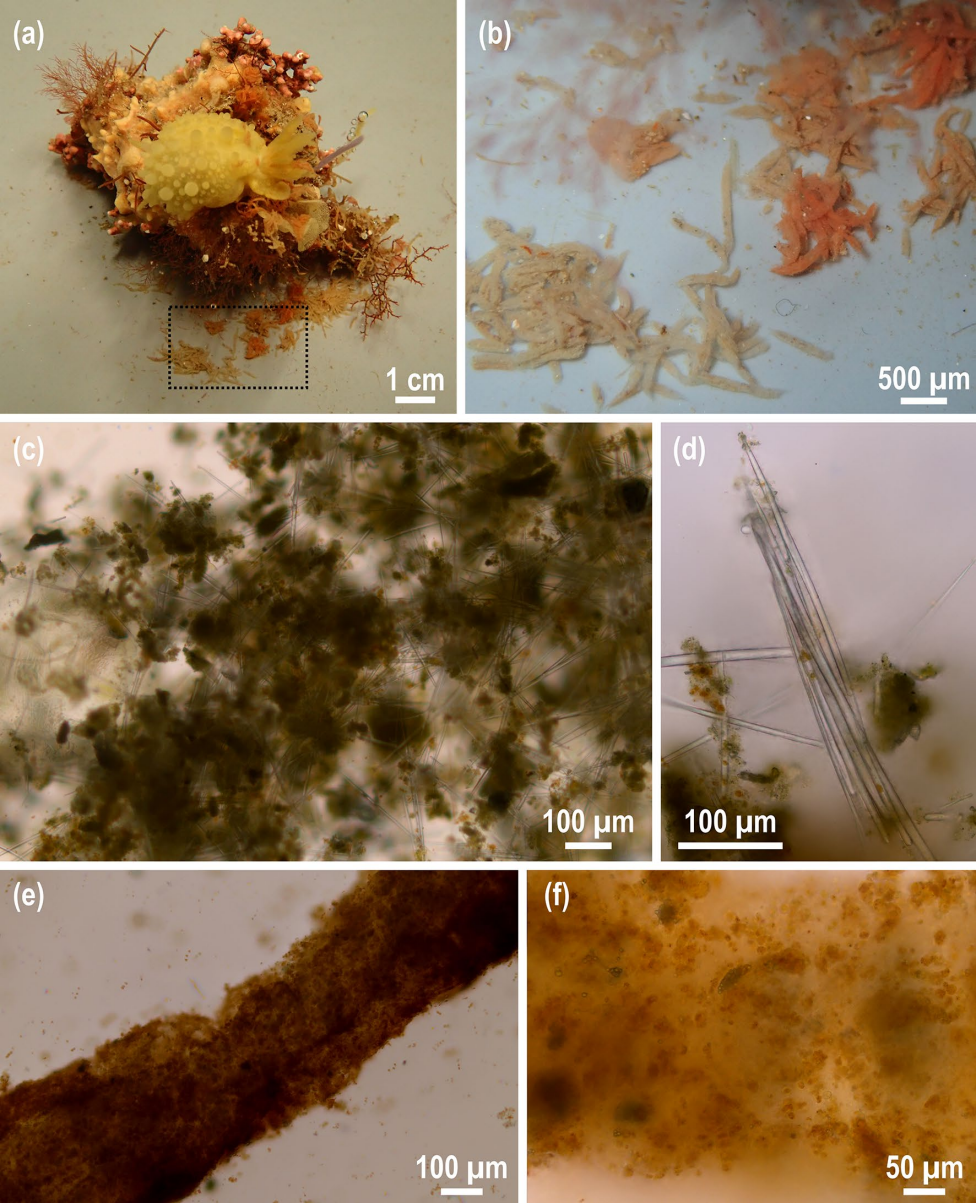
(**a**) The nudibranch *Doris verrucosa* feeds on the sponge *Hymeniacidon perlevis* after 72 h of aquarium incubation to assess the silicon export through nudibranch feces. (**b**) Enlargement of the dotted quadrat of (**a**), in which two types of fecal pellets are recognizable: colorless (left) and colored (right) pellets, the latter ones colored with the same pigments existing in the eaten sponge. (**c**, **d**) Microscopic view of colorless fecal pellets of *D. verrucosa*. Colorless feces, less structured than colored ones, are loaded with sponge spicules, which are mostly unbroken. In some cases (**d**), spicules still have the same structural organization as in the sponge. (**e**, **f**) Microscopic view of colored fecal pellets of *D. verrucosa*. Colored feces are more structured than colorless ones and mainly contain organic excretory debris.

Fecal production by nudibranchs was examined in the laboratory. The species *D. verrucosa* produced two types of feces when feeding on *H. perlevis* (Fig. 4a, b): colorless fecal pellets, which were unstructured and loaded with sponge spicules (Fig. 4c, d), and colored fecal pellets, which mostly lacked spicules and included organic excretory residues (Fig. 4e, f). Average fecal production was 11.6 ± 8.6 mg DW d^−1^ mL^−1^ nudibranch.

Silicon content in nudibranch feces ranged from 0.10 to 0.18 g Si g^−1^ DW, and the normalized rate of excretion ranged from 0.3 to 4.3 mg Si d^−1^ mL^−1^ nudibranch. In parallel, the amount of silicon ingested by each nudibranch was estimated by determining the amount of sponge eaten per nudibranch and the silicon content of each sponge examined. During laboratory experiments, nudibranchs ingested 0.4 to 4.1 mg Si d^−1^ mL^−1^ nudibranch. When comparing the amount of silicon ingested by each nudibranch with that found in feces, about 97.0 ± 8.7% of the ingested sponge silicon was excreted in nudibranch feces.

Based on the abundance of nudibranchs, the nudibranch predation rate and the specific silicon content of *H. perlevis*, the rate of sponge silicon deposition rate during predation was calculated. Nudibranchs ingested 1.2 ± 1.7 g Si m^−2^ during the period they were actively feeding (153 days, mid-June to mid-November). Using the ratio of sponge silicon excreted in feces (97.0 ± 8.7%), the rate of sponge silicon deposited through the feces of the nudibranch population during that period was estimated to be 1.2 ± 1.8 g Si m^−2^.

### Predated vs non-predated sponge silicon

To examine the effect of predation on sponge silicon, silicon fluxes and stocks of non-predated sponges were estimated and compared with those determined for predated sponges. Silicon production in non-predated sponges was estimated to be 0.3 ± 0.1 g Si m^−2^ from mid-June to mid-November and 0.7 ± 0.3 g Si m^−2^ from mid-November to mid-June. In those same periods, silicon deposition in non-predated sponges was estimated to be 0.2 ± 0.1 and 0.3 ± 0.2 g Si m^−2^, respectively. By adding the silicon production and subtracting the silicon deposition estimated from mid-June to mid-November to the measured silicon stock in June 2020, the silicon stock of non-predated sponges was estimated to be 2.7 ± 2.3 g Si m^−2^ in November 2020. By repeating the calculation for the period comprised between mid-November and mid-June, the silicon stock of non-predated sponges was estimated to be 3.1 ± 2.4 g Si m^−2^ in June 2021.

**Figure 5:**
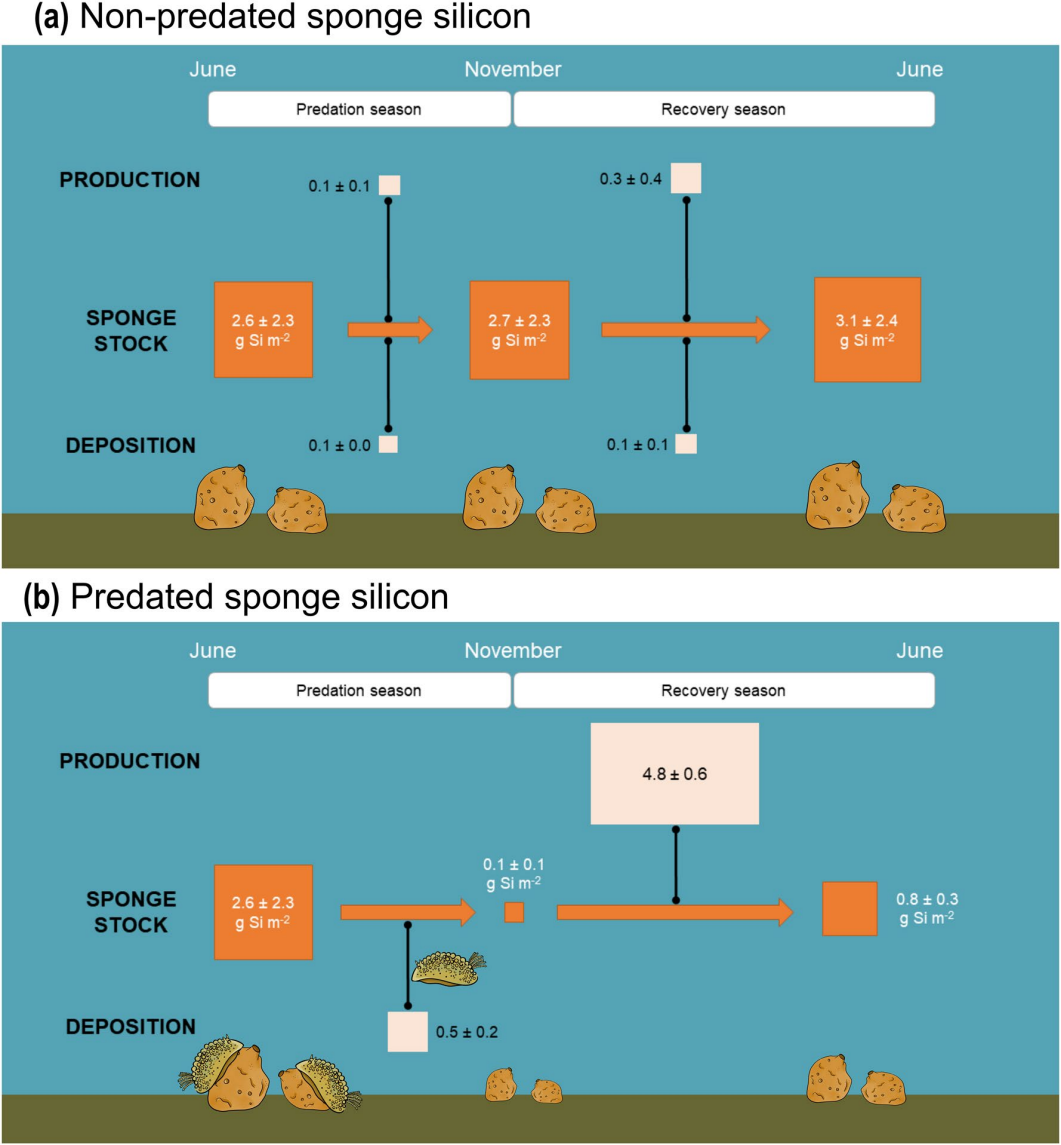
Scheme summarizing sponge silicon (Si) dynamics (**a**) as previously thought when predation is not considered and (**b**) integrating the predation effects measured in this study. The predation season of the nudibranch *Doris verrucosa*, which lasts from mid-June to mid-November, was determined from observations made during the survey of benthic fauna of the study area over the last decades (J. Grall, pers. comm.) and this study. Mean (± SD) sponge silicon stocks are indicated in g Si m^−2^ and stock-specific flux rates are dimensionless (g Si m^-2^ produced or deposited over the season considered : g Si m^−2^ of the initial sponge stock). The size of the boxes representing both stocks and stock-specific flux rates is proportional to their value. More details about the calculations are available in the main text and in Supplementary Information 1.

To compare silicon fluxes of predated and non-predated sponges, the stock-specific flux rates were calculated by normalizing the flux rate (either production or deposition) to the corresponding sponge silicon stock at the beginning of each study period. The rate of stock-specific sponge silicon deposition from mid-June 2020 to mid-November 2020 (i.e., when the spongivorous dorid nudibranch *D. verrucosa* was present) was 5 times faster than that estimated for non-predated sponges (Fig. 5), indicating that nudibranch predation boosts sponge silicon transport from the living community to the sediments below. From mid-November 2020 to mid-June 2021, the rate of stock-specific sponge silicon production was 16 times faster than that estimated for non-predated sponges (Fig. 5). This indicates that regenerating sponges consume silicon at much faster rates than undamaged sponges to recover their eaten biomass, mobilizing large amounts of silicon. Therefore, in a sponge population subjected to predation, sponge silicon deposition is the dominant flux during predation and sponge silicon production is the dominant flux during post-predation recovery (Fig. 5).

## Discussion

The sponge population survey of *Hymeniacidon perlevis* in the maerl bed of Lomergat (Bay of Brest, France) revealed remarkable changes over the course of a year. From June to November, the biomass of *H. perlevis* drastically decreased by 95 ± 1% (Fig. 3). That period coincides with the time when the nudibranch *Doris verrucosa* is present and feeds on the sponge *H. perlevis*, its only source of food reported in the area of study. At a feeding rate of 0.23 ± 0.11 mL sponge d^−1^ mL^−1^ nudibranch, the species *D. verrucosa* is responsible for 48 ± 33% of the sponge biomass decline. The rest of the sponge biomass lost could be due to the feeding activity of some facultative spongivores reported in the Bay of Brest, such as the polychaete *Euphrosine foliosa* and the gastropod *Emarginula fissure*^38^. There may be other causes for this biomass decline of *H. perlevis* such as sponge disease or mortality events^39^, although it is unlikely that these causes would have gone unnoticed during our periodic sampling.

During the predation season, deposition of sponge silicon in marine sediments increases as nudibranchs excrete most of the siliceous skeletons of the eaten sponges in the feces (97.0%; Fig. 4). This result is in agreement with other studies on strict spongivores such as hawksbill turtles, angelfishes, sea stars and other species of nudibranchs, in which high sponge silicon content in digestive tracts and feces have also been reported (68.3–99.9%)^18,19,26,40^. The increase of sponge silicon deposition will probably be more locally significant in areas where low-mobility spongivores, such as nudibranchs and sea stars, are present. The high deposition of sponge silicon enhances the role of sponges as silicon sinks. However, there is also the question of whether once sponge silica (opal) passes through the digestive tract of predators, it may become more susceptible to dissolution due to changes in pH or gastric proteolytic activity^41^. If so, this mechanism would contribute to increase dissolved silicon availability in demersal waters, which would help sponges regenerate eaten biomass.

Sponges usually recover their wounded tissue during the first month after damage, but when the damage is severe, the process can take several months^28,42^. In the study area, the number of sponges did not change during the year (Fig. 3a), but the average individual size decreased from 11.3 ± 20.3 to 0.7 ± 1.5 mL individual^−1^ from June to November, regardless of whether the decrease was due to the predation of *D. verrucosa* or another cause. After such severe biomass decrease, the sponge population recovered a total biomass of 69.7 ± 49.0 mL m^−2^, of which 27.7 ± 11.8 mL m^−2^ occurred from mid-November to mid-April and 42.0 ± 37.2 mL m^−2^ from mid-April to mid-June (Fig. 3b). The highest sponge growth (regeneration) measured in our study occurred when the availability of the main sponge food (pico- and nanoplankton, including bacteria, and dissolved organic matter) was at its highest, that is, from mid-April to June, when plankton blooms occurred^43^. Regeneration in sponges contrasts with the generally slow growth of these organisms^44^. Regeneration rates can be 2–10 times faster than growth rates^42,45^, and in some cases even up to 2900 times the natural growth rate^46^. Such high rates not only occur during regeneration but have also been measured in episodes of high availability of food in deep-sea and polar ecosystems, in which impressive sponge population explosions have been reported^47–49^.

To regenerate their damaged biomass, sponges must consume not only food to supply their energetic and growth needs but also dissolved silicon to build their siliceous skeletons as they grow. Unexpectedly, the highest silicon consumption occurred in the time of the year when dissolved silicon in seawater was minimal in the Bay of Brest (April-June; < 1.5 µM Si), when diatoms bloom and deplete dissolved silicon availability^50^. Furthermore, the estimated sponge silicon consumption from mid-April to mid-June during regeneration (0.4 ± 0.5 g Si m^−2^; Table 1) could not be fulfilled even if the concentration of dissolved silicon in seawater had been 100–150 µM Si, the concentration at which *H. perlevis* reaches its maximum silicon consumption rate^37,51^. This concentration has never been measured in the Bay of Brest and is only available in some remote zones such as the Southern Ocean and deep North Pacific Ocean^52^. In a hypothetical exercise, if *H. perlevis* would consume dissolved silicon at its maximum silicon consumption rate (V_max_ = 0.134 ± 0.018 µmol Si h^−1^ mL^−1^ sponge)^37^ from mid-April to mid-June, sponge growth would be 31.9 ± 12.1 mL m^−2^. This figure is calculated from the biomass measured in mid-April and integrates the daily growth over the 61 days up to mid-June (Supplementary Information 1). However, the sponge growth measured in the field at that period was 42.0 ± 37.2 mL m^−2^ (Table 1). The molecular and physiological mechanisms behind this high consumption of silicon are enigmatic. Silicon transport in sponges is partially due to passive transporters (aquaglyceroporins), which require a high dissolved silicon concentration outside the sponge to transport silicon within the sponge tissue, where silicon continues its transport and transformation for siliceous biomineralization of the sponge skeleton^53^. Our results suggest that unknown molecular, cellular or physiological mechanisms (or a combination of these) may be unlocked during regeneration to meet silicon requirements during sponge tissue regeneration. Gene expression analysis (including transcriptomics) of regenerating sponges could help to determine the genes involved in this process.

High exposure to waves, cold temperatures and/or high availability of dissolved silicon in seawater have been associated with seasonal variation in the inorganic:organic content of some intertidal and shallow-water sponges^54–56^, with seasonal variability not exceeding 10%. Our survey of the skeletal content of *H. perlevis* between June 2020 and June 2021 showed no significant differences between seasons. However, there was some between-individual variability in the skeletal content of *H. perlevis* that remains to be investigated to understand the factors (e.g., individual traits, environmental factors) responsible for skeletal variability between individuals of the same population.

The changes in sponge biomass measured in this study revealed the importance of intra- and inter-annual surveys for considering the effect of predation and sponge population dynamics in the study of sponge biogeochemistry and ecology. In the study area, sampling carried out at a single time to determine the annual sponge silicon budget would make errors of up to three orders of magnitude in both sponge silicon fluxes and stocks, depending on whether the sampling would be done in June or November, for instance, and it would omit sponge silicon fluxes that occur primarily in a given period of the year. In other regions such as the North Atlantic Ocean and the Southern Ocean, sponge biomass variations of 40–80% have also been attributed to predation and regeneration cycles over months to years^17,57^. This has implications for sponge monitoring planning, especially in the current scenario of anthropogenic climate change, in which marine ecosystems are changing rapidly and marine life is affected. Populations near the limit range of the species geographical distribution or under changing environmental conditions (e.g., ocean warming) can destabilize the predator–prey equilibrium^58,59^. An example of such a disruption has occurred on the coast of Alaska, where a heavy recruitment episode of the spongivore nudibranch *Archidoris montereyensis* led to the disappearance of the sponge species *Halichondria panicea* over an area of 550 square meters^60^. This loss led to a shift in the dominant species in the area, which is now the brown alga *Alaria marginata*^23^, causing changes in the biogeochemical cycling of silicon that have not been considered yet.

## Conclusion

Our results indicate that sponge predation not only modifies the structure and dynamics of sponge communities but also sponge-mediated biogeochemical cycling. They also show that intra-annual variation of predated sponge populations needs to be taken into account when measuring their silicon budget. Otherwise, biogeochemical estimates disconnected from the ecological dynamics can generate large errors in estimates of nutrient utilization. Our findings revealed unprecedented intra-annual changes in sponge silicon biomass, which were associated with higher rates of sponge silicon utilization than expected, irrespective of the dissolved silicon availability. This cycle of sponge predation-recovery at an annual rate suggests an enhanced nutrient cycling and has important implications for understanding the ecosystem functioning and the benthic-pelagic coupling of nutrients.

## Supplementary Information

Supplementary Information 1.

Supplementary Video S1.

## Data Availability

Metadata of this study are available at http://hdl.handle.net/10261/277857.
